# Knowledge and Adherence to Alendronate Administration Among Postmenopausal Osteoporosis Patients: A Cross-Sectional Study From a Tertiary Care Facility in Colombo South

**DOI:** 10.7759/cureus.96833

**Published:** 2025-11-14

**Authors:** Jenifar Prashanthan, Amirthanathan Prashanthan, Niranjala Meegoda Widanege, Chaminda Garusinghe

**Affiliations:** 1 Diabetes and Endocrinology, Colombo South Teaching Hospital, Colombo, LKA; 2 Applied Data and AI, Data Insights, Colombo, LKA; 3 Endocrinology, Colombo South Teaching Hospital, Colombo, LKA

**Keywords:** alendronate, bisphosphonates, drug-food interactions, medication adherence, multivariate analysis, osteoporosis, pharmaceutical care, postmenopausal women

## Abstract

Background

Compliance with alendronate treatment in postmenopausal women is inadequate due to intricate dosing protocols, resulting in diminished therapeutic effectiveness and heightened fracture risk. Although prior research has recorded adherence rates, few have determined independent determinants through multivariate analysis, especially within South Asian communities. This study sought to ascertain adherence rates, identify independent determinants of adherence, evaluate awareness of drug-food combinations, and examine the influence of various counseling sources on compliance.

Methods

A cross-sectional study was performed at Colombo South Teaching Hospital, Sri Lanka, from January to December 2024, involving 300 postmenopausal women diagnosed with osteoporosis who had been administered alendronate 70 mg weekly for a minimum duration of six months. The sample size was determined with the Lwanga and Lemeshow formula, presuming a 50% adherence rate, a 95% confidence interval, and a 5.7% margin of error. A validated questionnaire evaluated compliance with seven dosing instructions, understanding of 13 drug-food-medication combinations, side effects, and sources of patient education. Multivariate logistic regression discovered independent determinants of optimal adherence.

Results

The average age was 67.4±8.9 years. Only 48 participants (16.0%) attained optimal adherence (compliance score ≥6/7), with a median compliance score of 4.0 (IQR: 3.0-5.0). In multivariate analysis, the independent predictors of optimal adherence included pharmacist counseling (adjusted OR=3.24, 95% CI: 1.87-5.61, p<0.001), comprehensive understanding of instructions (adjusted OR=2.89, 95% CI: 1.65-5.06, p<0.001), tertiary education (adjusted OR=2.15, 95% CI: 1.23-3.76, p=0.007), and suburban residence (adjusted OR=1.87, 95% CI: 1.12-3.14, p=0.017). The median knowledge score was 6.0 out of 13, with an interquartile range of 5.0 to 7.0. Significant knowledge deficiencies encompassed erroneous notions regarding co-administration with liquids other than plain water (154 participants, 51.3%). Adverse effects were reported by 187 individuals (62.3%), primarily gastrointestinal problems.

Conclusions

Compliance with alendronate is significantly low among postmenopausal women in Sri Lanka. Pharmacist advice and comprehensive comprehension of instructions were identified as the most significant modifiable determinants of adherence. Healthcare systems must establish organized pharmaceutical care programs and create instructional materials suitable for health literacy to enhance osteoporosis treatment results.

## Introduction

Osteoporosis is a degenerative skeletal condition marked by diminished bone mineral density and microarchitectural degradation, resulting in heightened fracture risk [1,2]. The illness disproportionately impacts postmenopausal women, with a global prevalence estimated at 30-50% among those over 50 years of age [3]. Fractures of the hip, vertebrae, and wrist represent the principal clinical outcomes, leading to considerable morbidity, mortality, diminished quality of life, and large healthcare expenditures.

Alendronate, a nitrogenous bisphosphonate, constitutes the primary pharmaceutical treatment for postmenopausal osteoporosis [4]. Randomized controlled trials have shown that alendronate decreases the risk of vertebral fractures by 47% and non-vertebral fractures by 51% when taken correctly [5]. Therapeutic efficacy is fundamentally reliant on rigorous compliance with intricate dosing protocols: administration alone with plain water, in a fasting condition, succeeded by keeping an upright position for a minimum of 30 minutes prior to the intake of food or other medications [6].

Inadequate compliance with bisphosphonate medication constitutes a significant obstacle to effective osteoporosis care. Prior research has recorded dropout rates ranging from 19% to 56% throughout the initial year of treatment [7,8]. Inadequate adherence correlates with diminished therapy effectiveness, elevated fracture risk, increased healthcare expenditures, and heightened morbidity [9,10]. Moreover, gastrointestinal side effects, observed in 15-30% of users, lead to treatment termination and non-compliance [11].

Numerous factors affect medication adherence, such as intricate dose schedules, insufficient patient education, side effects, socioeconomic status, and health literacy levels [12,13]. Although prior research has descriptively recorded adherence rates, limited studies have utilized multivariate statistical techniques to ascertain independent predictors of non-adherence while accounting for confounding variables. Furthermore, the majority of current material is derived from Western cultures, with scant data from South Asian nations, where healthcare systems, literacy rates, patient education methods, and cultural influences vary significantly.

Comprehending independent predictors of adherence is crucial for formulating tailored, evidence-based treatments to enhance treatment results. Recognizing modifiable factors, especially the origin and quality of patient counseling, may lead to health system policy and clinical practice recommendations to enhance osteoporosis care. This study aims to ascertain the prevalence of adherence to alendronate dosage guidelines among postmenopausal women diagnosed with osteoporosis, identify independent predictors of optimal compliance through multivariate logistic regression analysis, evaluate patient understanding of interactions between alendronate and food or other medications, assess the impact of various counseling sources on adherence outcomes, document the frequency and categories of adverse effects experienced by patients, and analyze the correlation between the level of comprehension of instructions and adherence to therapy.

This study advances existing knowledge by performing the first multivariate analysis of factors influencing alendronate adherence in a South Asian population, assessing the unique independent effect of pharmacist counseling relative to other information sources, investigating the relationship between comprehension levels and adherence outcomes while considering educational background, utilizing an adequately powered sample size for multivariate analysis and subgroup comparisons, and comprehensively examining various domains including adherence behaviors, knowledge, adverse effects, and educational programs.

## Materials and methods

Research design and context

This hospital-based cross-sectional study was performed in the Diabetes and Endocrinology Outpatient Clinic of Colombo South Teaching Hospital, a tertiary care facility situated in Kalubowila, Sri Lanka. The hospital caters to a catchment population of roughly 1.2 million in the Colombo South area, encompassing the Dehiwala-Mount Lavinia Municipal Council, Moratuwa Municipal Council, and portions of Colombo District (South) and Kalutara District. The population characteristics include mixed urban, suburban, and rural areas with a socioeconomically diverse population that is representative of the general Sri Lankan postmenopausal population. Data gathering occurred during a 12-month span from January 2024 to December 2024. This is an observational cross-sectional study. No educational intervention was provided to participants. We assessed existing knowledge and adherence patterns, including evaluation of instructions previously received from healthcare providers, media, or other sources.

Determination of sample size

Sample size was calculated prospectively using the formula proposed by Lwanga and Lemeshow [14] for estimating population proportions:

 $n = \frac{Z^2 \times p \times (1-p)}{d^2}$

where n represents the required sample size, Z equals 1.96 (standard normal deviate at 95% confidence level), p equals 0.50 (expected proportion of adherent patients; 50% provides maximum sample size and was chosen based on previous studies reporting adherence rates ranging from 40 to 60%), and d equals 0.057 (desired precision/margin of error).

Calculation:

n = [(1.96)² × 0.50 × 0.50] / (0.057)²

n = [3.8416 × 0.25] / 0.00325

n = 0.9604 / 0.00325

n = 295.5 ≈ 296

We enrolled 300 participants, adding a 1.4% contingency (n=4) to account for potential incomplete responses or data entry errors. The calculated sample size (296) and the final enrollment (300) are explicitly stated to provide transparency in sampling methodology.

Study cohort and sampling methodology

The study comprised postmenopausal women aged 45 years or older, with menopause characterized as the cessation of menstruation for a minimum of 12 consecutive months. Participants needed a verified diagnosis of postmenopausal osteoporosis, indicated by a bone mineral density T-score of -2.5 or lower at any site assessed via dual-energy X-ray absorptiometry, or a history of fragility fracture characterized as a low-trauma fracture occurring from standing height or less. All participants possessed a current prescription for alendronate sodium 70 mg weekly without treatment discontinuation and had undergone continuous prescription (not discontinued by a physician) for a minimum of six months at enrollment at the time of registration. Further prerequisites encompassed the capacity to comprehend and address questionnaire items in Sinhala, Tamil, or English, alongside the giving of signed informed permission.

Patients were excluded if they exhibited cognitive impairment that hindered accurate questionnaire completion, had a treatment duration of less than six months, or were concurrently using other bisphosphonates or bone-active medications, including teriparatide, denosumab, raloxifene, or hormone replacement therapy; however, calcium and vitamin D supplements were allowed. Patients with significant comorbidities that hindered their communication abilities were also excluded. Consecutive sampling was utilized over the whole study duration. All qualified patients visiting the clinic were solicited to participate sequentially until the desired sample size was reached. Out of 324 eligible patients approached, 300 provided consent and completed the trial, resulting in a response rate of 92.6%. Twenty-four patients refused to participate, with 18 attributing their decision to time restrictions and six declining without specifying reasons.

Data collection tool

A systematic questionnaire was created utilizing validated items from existing studies [13,15,16] and tailored to the Sri Lankan context via an extensive validation process. Content validation entailed an expert review conducted by three consultant endocrinologists and two clinical pharmacists, who evaluated the instrument's relevance, intelligibility, and cultural appropriateness through iterative revisions until consensus was reached. The questionnaire was translated into Sinhala and Tamil by forward-backward translation by licensed medical translators, thereafter reviewed by multilingual healthcare specialists, and pilot tested to ensure language clarity. A pilot study was performed with 20 postmenopausal women, who were excluded from the final sample, facilitating adjustments based on input on comprehension. The mean completion time was determined to be between 20 and 25 minutes. The reliability assessment indicated satisfactory internal consistency for the knowledge section, with a Cronbach's alpha of 0.78, and strong internal consistency for the compliance section, with a Cronbach's alpha of 0.82. The final questionnaire consisted of four sections containing a total of 34 items, as outlined in Appendix 1.

Data collection methodology

Two trained research assistants, both registered nurses with research expertise, conducted structured face-to-face interviews in private consultation rooms. The research assistants received extensive training comprising eight hours of instruction on informed consent procedures, questionnaire administration, and neutral interviewing approaches. This training included standardized question-reading protocols requiring questions to be read verbatim without paraphrasing, practice sessions with feedback to ensure consistency across interviewers, and comprehensive instruction on data quality and confidentiality protocols. Throughout the data collection period, weekly meetings were held to address emerging questions and maintain standardization among the research team.

To ensure inter-rater reliability, 10% of interviews (n=30) were audio-recorded with participant consent and subsequently reviewed for consistency. Questions were read verbatim from the structured questionnaire, and response options were standardized using multiple-choice and Likert scales. Any ambiguous responses were flagged for investigator review to ensure accurate data capture and interpretation.

Participants chose their preferred language from among Sinhala, Tamil, and English. Research assistants orally presented questions and documented replies on paper forms. This interviewer-administered method was developed to address diverse reading levels and guarantee understanding among all participants, ensuring accessibility regardless of literacy level.

While the potential for interviewer bias is acknowledged, several measures were implemented to minimize inconsistency between interviewers. These included the standardized training program, ongoing monitoring protocols, and the use of structured instruments with verbatim reading of questions, all designed to reduce variability and enhance the reliability and validity of the collected data.

Assurance of data quality

Various quality control techniques were instituted to guarantee data integrity. The measures encompassed daily assessments of finalized surveys, dual data entry by separate staff, range and logic validations to detect discrepancies, and random verification of 10% of all entries. The interviewer-administered format with real-time completeness verification yielded no missing data.

Statistical evaluation

Data were inputted into Microsoft Excel (Redmond, USA) and analyzed utilizing R version 4.3.1 and RStudio software. Categorical variables were summarized by frequencies and percentages. The Kolmogorov-Smirnov test was employed to evaluate the distribution of continuous variables, with statistical reporting guidelines applied based on data distribution patterns. For normally distributed continuous variables (Kolmogorov-Smirnov p>0.05), data are reported as means with standard deviations only. For non-normally distributed continuous variables (p<0.001), data are conveyed as medians with interquartile ranges (25th to 75th percentiles) as the primary measure, with mean ± SD provided as supplementary information to aid comparison with studies that reported only means.

Normality testing revealed that age, osteoporosis duration, and alendronate duration were normally distributed (p=0.132, p=0.089, and p=0.156, respectively) and were therefore reported as mean ± SD only. In contrast, compliance scores and knowledge scores demonstrated non-normal distribution with p-values below 0.001, necessitating the use of median with interquartile range as the primary measure and mean ± SD as supplementary information. Due to the non-normal distribution of compliance and knowledge ratings, non-parametric statistical tests were required for subsequent analyses.

Bivariate analyses were performed utilizing suitable statistical tests according to the types and distributions of the variables. The Mann-Whitney U test was utilized to compare scores between two groups, whereas the Kruskal-Wallis test was applied to compare scores among three or more groups. Chi-square or Fisher's exact tests were employed to analyze relationships between categorical variables. Upon obtaining significant findings from the Kruskal-Wallis test, post-hoc Dunn's tests with Bonferroni correction were conducted for pairwise comparisons to identify specific group differences.

Binary logistic regression was used to ascertain independent predictors of ideal adherence, with the dependent variable defined as a compliance score of six or above versus less than six. The model construction procedure encompassed multiple stages. Initially, factors with p-values below 0.20 in bivariate analysis or those considered clinically significant were selected as candidates for inclusion. Multicollinearity was evaluated by variance inflation factors, with acceptable values required to be below five. Backward stepwise elimination was utilized to exclude variables with p-values of 0.05 or more, resulting in a final model that retained only significant predictors with p-values below 0.05.

Model diagnostics comprised the Hosmer-Lemeshow test for goodness of fit, where p-values beyond 0.05 signify a satisfactory model fit, and the area under the receiver operating characteristic (ROC) curve to assess discriminative capability, with values surpassing 0.70 being acceptable. Classification accuracy, sensitivity, and specificity were computed to evaluate model performance. Results were presented as adjusted odds ratios accompanied by 95% confidence intervals. The threshold for statistical significance was established at a two-tailed p-value of under 0.05 for all analyses.

Ethical considerations

The research obtained authorization from the Ethical Review Committee of Colombo South Teaching Hospital (Approval Number: IERC/2036, dated 09/01/2024) and complied with the standards established in the Declaration of Helsinki. Informed consent was acquired in writing from all participants in their preferred language: Sinhala, Tamil, or English. Before gaining consent, participants were provided with comprehensive descriptions of the study's objectives and procedures, the voluntary nature of their involvement, their right to withdraw at any time without impacting their medical care, and the confidentiality measures implemented.

To ensure truly voluntary consent, several safeguards were implemented throughout the recruitment process. Recruitment occurred separately from clinical consultations, and treating physicians were not involved in the consent process to avoid any perception of coercion. Participants were explicitly informed that the research team was independent from their clinical care team and that their participation or refusal would not affect their medical care or relationship with healthcare providers in any way. Participants were given adequate time for questions and consideration before signing consent forms and were informed of their right to withdraw at any point without providing reasons and without any consequences. No compensation or incentives were provided to participants to avoid coercion and guarantee genuinely voluntary involvement.

Thorough data protection protocols were adopted during the investigation. Every participant received a distinct identification number, and personal identifiers were maintained separately from study data in password-protected files. Data were disclosed solely in aggregate form to avert the identification of individual participants. All research data will be preserved for five years in compliance with institutional policy. The study presented negligible risk to participants, requiring merely a time commitment of 20 to 25 minutes for questionnaire completion, with no physical dangers connected with participation.

## Results

Sociodemographic and clinical characteristics

The study had 300 participants with a mean age of 67.4±8.9 years, predominantly within the 65-74 year age range (113 participants, 37.7%). The majority had primary or secondary education (88 participants, 29.1%), while more than one-quarter held tertiary qualifications (80 participants, 26.8%). The income distribution was rather equitable across the low (99 participants, 33.1%), intermediate (102 participants, 33.9%), and high (83 participants, 27.6%) groups. The predominant number of participants resided in suburban areas (126 participants, 42.1%) and were unemployed (175 participants, 58.3%).

All participants were lifetime non-smokers who had never used tobacco products, which is consistent with the lifestyle patterns of postmenopausal women in Sri Lanka, where female smoking prevalence is very low at less than one percent according to national surveys. Slightly over half of the participants consumed alcohol (163 participants, 54.2%), while fewer than half participated in regular physical activity (145 participants, 48.3%).

Body mass index was evenly distributed throughout categories, with equal proportions of participants classified as underweight and normal weight (70 participants each, 23.3%) and as overweight and obese (80 participants each, 26.7%). Body mass index (BMI) was assessed through standardized measurements, with weight measured using a calibrated digital scale to the nearest 0.1 kg and height measured using a stadiometer to the nearest 0.5 cm. BMI was calculated as weight in kilograms divided by height in meters squared. Categories were based on the World Health Organization (WHO) international classification, with underweight defined as less than 18.5 kg/m², normal weight as 18.5-24.9 kg/m², overweight as 25.0-29.9 kg/m², and obese as 30.0 kg/m² or greater. While WHO international BMI cut-points were used rather than Asian-specific thresholds, which may not optimally represent metabolic risk in this population, the analysis showed no significant association between BMI and adherence outcomes.

In the clinical study, 125 individuals (41.7%) indicated a history of fractures, with an average osteoporosis duration of 4.2±2.8 years and an average alendronate therapy duration of 18.6±10.2 months. Table 1 delineates the sociodemographic and clinical characteristics of the 300 participants.

**Table 1:** Sociodemographic and clinical characteristics of participants (N=300) BMI: Body Mass Index, calculated from measured height and weight; categorized per WHO international classification

Characteristic	Category	Value	n	%
Age	Mean ± SD	67.4 ± 8.9 years	-	-
	Range	48-87 years	-	-
	45-54 years	-	21	7.0
	55-64 years	-	100	33.3
	65-74 years	-	113	37.7
	≥75 years	-	66	22.0
Education	Literate	-	45	14.9
	Primary	-	36	11.9
	Secondary	-	52	17.2
	Diploma	-	56	18.7
	Bachelor's	-	40	13.4
	Postgraduate	-	40	13.4
	Tertiary education (total)	-	-	26.8
Income	Low	-	99	33.1
	Moderate	-	102	33.9
	High	-	83	27.6
Residence	Rural	-	88	29.4
	Suburban	-	126	42.1
	Urban	-	71	23.8
Employment	Unemployed	-	175	58.3
	Employed	-	125	41.7
Lifestyle	Non-smokers	-	300	100.0
	Alcohol use	-	163	54.2
	Physical activity	-	145	48.3
BMI	Underweight (<18.5)	-	70	23.3
	Normal (18.5-24.9)	-	70	23.3
	Overweight (25.0-29.9)	-	80	26.7
	Obese (≥30.0)	-	80	26.7
Clinical	Fracture history	-	125	41.7
	Osteoporosis duration	4.2 ± 2.8 years	-	-
	Alendronate duration	18.6 ± 10.2 months	-	-

Adherence to dosing instructions

Adherence rates for individual items varied from 130 participants (43.3%) to 170 participants (56.7%), with the highest compliance noted for medication timing criteria (avoiding other medications for ≥30 minutes, 170 participants, 56.7%) and food timing (≥30 minutes before eating, 165 participants, 55.0%). The lowest adherence rates were seen for plain water drinking (130 participants, 43.3%) and a weekly dosage regimen (135 participants, 45.0%).

The finding that only 45% followed the weekly dosing schedule correctly demonstrates an important methodological distinction: our inclusion criterion of "continuous treatment for ≥6 months" referred to continuous prescribing by physicians (prescriptions were not discontinued), not to patient adherence. Patients may collect prescriptions regularly while failing to adhere to complex administration protocols. This finding highlight that continuous prescribing does not guarantee adherent behavior.

The median overall compliance score was 4.0 (IQR: 3.0-5.0) out of a maximum of 7, with a mean of 3.8 ± 1.6. The majority of individuals demonstrated moderate adherence (193 participants, 64.3%), while only 48 participants (16.0%) attained good adherence (6-7 items right), and a paltry six participants (2.0%) exhibited perfect adherence to all seven dose parameters. Approximately 59 participants (19.7%) demonstrated inadequate adherence by accurately fulfilling two or fewer administration requirements. Table 2 presents adherence scores by item and overall compliance metrics (N=300).

**Table 2:** Item-specific adherence and overall compliance scores (N=300)

Adherence Component	Category/Item	n	%	Score
Item-Specific Adherence	1. Weekly dosing schedule	135	45.0	-
	2. Morning administration	150	50.0	-
	3. Plain water	130	43.3	-
	4. Upright posture 30 min	152	50.8	-
	5. Missed dose protocol	150	50.0	-
	6. Food timing ≥30 min	165	55.0	-
	7. Medication timing ≥30 min	170	56.7	-
Overall Compliance Scores	Median (IQR)	-	-	4.0 (3.0-5.0)
	Mean ± SD	-	-	3.8 ± 1.6
	Poor adherence (0-2)	59	19.7	-
	Partial adherence (3-5)	193	64.3	-
	Optimal adherence (6-7)	48	16.0	-
	Perfect adherence (7/7)	6	2.0	-

Sources and understanding of instructions

Just over half of the participants (157 participants, 52.3%) reported receiving administration instructions from any source, while 143 people (47.7%) either did not receive instructions or could not recall acquiring them. Of the 143 participants (47.7%) who reported not receiving instructions or could not recall, an unknown proportion may have actually received instructions but forgotten them. This misclassification bias would lead to underestimation of true instruction coverage and potential overestimation of the pharmacist counseling effect if those who recalled were systematically different from those who forgot. This represents a crossover between exposure groups (received vs. not received instructions) and is inherent to retrospective self-report.

Of the 157 individuals who received instructions, the primary sources were media (38 participants, 24.2%) and unidentified sources (38 participants, 24.2%), followed by pamphlets (31 participants, 20.0%), pharmacies (26 participants, 16.7%), and physicians (24 participants, 15.0%). Instructions were distributed uniformly in Sinhala and Tamil (52 participants each, 33.3%), followed by English (45 participants, 28.6%) and mixed languages (8 participants, 5.1%). Regarding comprehension, slightly more than one-third (56 participants, 35.8%) showed complete understanding of all instructions, while 62 participants (39.2%) exhibited partial understanding, and 39 participants (25.0%) displayed minimal understanding.

These understanding levels (complete/partial/minimal) were participant self-assessments, not researcher classifications. Participants were asked, "How well did you understand the instructions?" with standardized response options: "All (complete understanding)" / "Some (partial understanding)" / "Little (minimal understanding)." While standardized comprehension tests would be ideal, self-reported understanding has been used in prior adherence studies and has predictive validity for actual adherence behavior (as demonstrated in our multivariate analysis). However, understanding levels were based on self-assessment rather than objective comprehension testing, which may be subject to overestimation due to social desirability bias or metacognitive limitations.

These findings reveal significant shortcomings in the provision and comprehension of essential prescription administration directives. Table 3 presents the receipt, sources, language, and comprehension of medication instructions.

**Table 3:** Receipt, sources, language, and understanding of medication instructions (N=300) *Among those receiving instructions (n = 157)

Instruction Component	Category	n	%
Receipt of Instructions	Received from any source	157	52.3
	Did not receive or cannot recall	143	47.7
Sources*	Media	38	24.2
	Leaflets	31	20.0
	Pharmacist	26	16.7
	Doctor	24	15.0
	Unknown	38	24.2
Language*	Sinhala	52	33.3
	Tamil	52	33.3
	English	45	28.6
	Mixed	8	5.1
Understanding Level*	All (complete)	56	35.8
	Some (partial)	62	39.2
	Little (minimal)	39	25.0

Knowledge of drug-food interactions

The median knowledge score was 6.0 (IQR: 5.0-7.0) out of a maximum of 13 points, with a mean of 6.2±2.3, indicating moderate general knowledge. The response rates for individual items ranged from 128 participants (42.5%) to 155 participants (51.7%), reflecting consistently insufficient knowledge across all assessed categories. The highest accurate response rate related to tap water usage (155 participants, 51.7%), suggesting that nearly half of the participants were unaware of this fundamental necessity.

The comprehension of pharmacological interactions was significantly deficient, with just 152 individuals (50.8%) correctly identifying cortisone-related concerns and 150 participants (50.0%) aware of NSAID interactions. Dietary knowledge exhibited similar deficits, with less than half correctly answering questions about green vegetables (148 participants, 49.2%), dairy products (137 participants, 45.8%), and calcium supplements (133 participants, 44.2%).

Substantial knowledge gaps were evident, as 145 participants (48.3%) mistakenly thought that beverages other than tap water were appropriate for prescription delivery, and 167 participants (55.8%) were unaware that calcium supplements should be taken separately from the alendronate dose. These findings highlight substantial knowledge gaps that likely result in improper administration and insufficient adherence. Table 4 presents knowledge scores and correct response rates for individual knowledge items.

**Table 4:** Knowledge scores and correct response rates for individual knowledge items (N=300)

Knowledge Assessment	Item/Category	n	%	Score
Knowledge Scores	Median (IQR)	-	-	6.0 (5.0-7.0)
	Mean ± SD	-	-	6.2 ± 2.3
Correct Response Rates	1. Tap water	155	51.7	-
	2. Cortisone	152	50.8	-
	3. Vitamin D	150	50.0	-
	4. NSAIDs	150	50.0	-
	5. Leafy vegetables	148	49.2	-
	6. Coffee	145	48.3	-
	7. Mineral water	143	47.5	-
	8. Milk	143	47.5	-
	9. Dairy products	137	45.8	-
	10. Tea	133	44.2	-
	11. Calcium supplements	133	44.2	-
	12. Antacids	130	43.3	-
	13. Fruit juices	128	42.5	-

Bivariate analysis

Table 5 presents a comprehensive bivariate analysis of the relationship between sociodemographic characteristics and the compliance and knowledge ratings of the study participants. The analysis employed non-parametric statistical tests, specifically the Kruskal-Wallis test for variables with multiple categories and the Mann-Whitney U test for binary variables, to assess the significance of associations. This approach was necessitated by the non-normal distribution of the data, as evidenced by the use of medians and interquartile ranges. No statistically significant correlations were identified between compliance or knowledge scores and any of the examined sociodemographic variables, since all p-values exceeded the 0.05 significance level.

**Table 5:** Bivariate analysis compliance and knowledge scores by sociodemographic variables (N=300) H: Kruskal-Wallis test statistic; U: Mann-Whitney U test statistic; *p<0.05 considered statistically significant

Variable	Category	Compliance Score Median (IQR)	Test Statistic	P-value	Knowledge Score Median (IQR)	Test Statistic	P-value
Age	45-54	4.0 (3.8-5.0)	H=4.76	0.193	6.5 (5.8-8.0)	H=1.90	0.592
	55-64	3.0 (2.0-4.0)	-	-	6.0 (5.0-7.0)	-	-
	65-74	3.0 (2.5-4.0)	-	-	6.0 (5.0-8.0)	-	-
	≥75	4.0 (3.0-5.0)	-	-	6.0 (5.0-7.0)	-	-
Education	Literate	3.5 (2.0-5.0)	H=5.52	0.341	6.0 (5.0-7.0)	H=3.78	0.606
	Primary	3.0 (2.0-4.0)	-	-	6.0 (5.0-7.0)	-	-
	Secondary	4.0 (3.0-4.5)	-	-	6.0 (4.5-8.0)	-	-
	Diploma	4.0 (3.0-5.0)	-	-	6.0 (5.0-7.0)	-	-
	Bachelor's	3.0 (2.0-4.0)	-	-	5.0 (5.0-7.8)	-	-
	Postgraduate	3.5 (3.0-4.8)	-	-	7.0 (6.0-7.8)	-	-
Income	Low	4.0 (3.0-4.8)	H=0.58	0.748	6.0 (5.0-7.0)	H=0.84	0.658
	Moderate	4.0 (2.0-4.0)	-	-	6.0 (5.0-7.5)	-	-
	High	3.0 (2.5-5.0)	-	-	5.0 (5.0-7.0)	-	-
Residence	Rural	4.0 (2.0-4.0)	H=1.07	0.587	6.0 (5.0-7.0)	H=1.31	0.518
	Suburban	4.0 (3.0-4.0)	-	-	6.0 (5.0-8.0)	-	-
	Urban	3.0 (2.0-4.0)	-	-	6.0 (5.0-7.0)	-	-
Employment	Employed	4.0 (2.2-4.8)	U=9875	0.498	6.0 (5.0-7.0)	U=9542	0.275
	Unemployed	3.0 (2.2-4.0)	-	-	6.0 (5.0-8.0)	-	-
Physical Activity	Yes	4.0 (2.2-5.0)	U=10234	0.621	6.0 (5.0-7.0)	U=9321	0.139
	No	3.5 (2.2-4.0)	-	-	6.0 (5.0-7.0)	-	-
BMI	<18.5	3.5 (2.0-5.0)	H=2.66	0.449	5.5 (4.8-7.0)	H=3.38	0.337
	18.5-24.9	4.0 (3.0-5.0)	-	-	6.0 (5.0-7.2)	-	-
	25.0-29.9	3.0 (2.0-4.0)	-	-	6.0 (5.0-7.0)	-	-
	≥30.0	4.0 (2.8-5.0)	-	-	7.0 (5.0-8.0)	-	-

Despite these null findings, Table 5 serves important purposes in transparent reporting and contributes meaningfully to scientific literature. The comprehensive reporting of non-significant results demonstrates which variables were not significant, thereby preventing publication bias that often favors positive findings. It shows that potential confounders were assessed systematically and allows readers to evaluate what was tested before multivariate selection. This transparency about null findings is crucial for enabling meta-analyses and future research planning, as it provides valuable information about which demographic factors do not confound the instruction-adherence relationship. Such reporting ensures that future researchers can build upon this knowledge and avoid redundant investigations.

Notably, physical activity showed no significant association with compliance (p=0.621) or knowledge (p=0.139), suggesting that educational interventions may be needed regardless of baseline health behaviors. This finding indicates that health-promoting behaviors in one domain do not necessarily translate into better medication adherence or disease knowledge in another, highlighting the importance of targeted educational interventions for all patients regardless of their lifestyle characteristics.

Multivariate logistic regression: independent predictors

Multivariate logistic regression identified four independent variables of optimal adherence. Pharmacist advice was identified as the primary predictor, increasing the probability of optimal adherence by more than threefold compared to the lack of guidance (adjusted OR=3.24, 95% CI: 1.87-5.61, p<0.001). A thorough comprehension of instructions markedly enhanced the probability of optimal adherence relative to limited knowledge (adjusted OR=2.89, 95% CI: 1.65-5.06, p<0.001). Tertiary education markedly enhanced the likelihood relative to primary education or literacy alone (adjusted OR=2.15, 95% CI: 1.23-3.76, p=0.007). Furthermore, suburban residency increased the likelihood by 87% compared to rural residency (adjusted OR=1.87, 95% CI: 1.12-3.14, p=0.017). Table 6 displays a multivariate logistic regression analysis identifying independent determinants of excellent adherence.

**Table 6:** Multivariate logistic regression analysis independent predictors of optimal adherence (score ≥6) (N=300) OR: Odds Ratio; CI: Confidence Interval; Wald χ²: Wald chi-square statistic from binary logistic regression; *p<0.05 considered statistically significant

Predictor	Comparison	Adjusted OR	95% CI	Wald χ²	P-value
Pharmacist counseling	vs. no instructions	3.24	1.87-5.61	18.45	<0.001*
Complete understanding	vs. minimal	2.89	1.65-5.06	15.32	<0.001*
Tertiary education	vs. primary/literacy	2.15	1.23-3.76	7.28	0.007*
Suburban residence	vs. rural	1.87	1.12-3.14	5.67	0.017*

The resulting multivariate model demonstrated robust statistical performance across multiple diagnostic metrics. The Hosmer-Lemeshow goodness-of-fit test indicated satisfactory model calibration (p=0.42), signifying no substantial discrepancy between observed and projected values. The model exhibited robust discriminative capability, as indicated by an AUC-ROC of 0.76 (95% CI: 0.70-0.82), reflecting a commendable ability to distinguish between individuals with good and inadequate adherence. The overall classification accuracy was 71.3%, signifying that the model correctly classified about 7 of 10 participants on their adherence status. Table 7 displays the diagnostics for the logistic regression model.

**Table 7:** Logistic regression model diagnostics (N=300) AUC-ROC: Area Under the Receiver Operating Characteristic Curve

Model Diagnostics	Value	95% CI	Interpretation
Hosmer-Lemeshow test	p = 0.42	-	Good fit
AUC-ROC	0.76	0.70-0.82	Good discrimination
Classification accuracy	71.3%	-	-

Adverse effects

Approximately 187 participants (62.3%) reported experiencing at least one adverse event associated with alendronate medication. Gastrointestinal issues constituted the predominant adverse effects, with peptic ulcer documented in 164 instances (54.7%), followed by gastritis at 160 instances (53.3%). Other common reactions included dysphagia or gingival edema (145 participants, 48.3%), dyspepsia (143 participants, 47.5%), and general gastrointestinal intolerance (130 participants, 43.3%). Hemoptysis was reported by 128 participants (42.5%), a figure that is unexpectedly elevated and may suggest problems with symptom misclassification or misattribution.

Regarding severity distribution, 113 people (37.7%) reported no side effects, whereas the majority exhibited mild (86 participants, 28.7%) or moderate (73 participants, 24.3%) symptoms. Twenty-eight subjects (9.3%) indicated experiencing serious side effects. The notable incidence of side effects, particularly gastrointestinal problems, is likely attributable to a confluence of incorrect administration practices, recall bias, pre-existing diseases, and possibly misattribution of unrelated symptoms to alendronate therapy. Table 8 illustrates the occurrence and severity distribution of side effects.

**Table 8:** Adverse effect prevalence and severity distribution (N=300) GI: Gastrointestinal

Adverse Effect	Category/Type	n	%
Overall	Any adverse effect	187	62.3
Specific Effects	1. Peptic ulcer	164	54.7
	2. Gastritis	160	53.3
	3. Dysphagia/gingival swelling	145	48.3
	4. Dyspepsia	143	47.5
	5. GI intolerance	130	43.3
	6. Hemoptysis	128	42.5
Severity Distribution	None	113	37.7
	Mild	86	28.7
	Moderate	73	24.3
	Severe	28	9.3

## Discussion

Principal findings

This cross-sectional study involving 300 postmenopausal women demonstrated very low compliance with alendronate dose guidelines, with just 48 participants (16%) exhibiting excellent adherence and a mere six participants (2%) achieving perfect adherence. Multivariate analysis revealed four independent predictors: pharmacist counseling (OR=3.24), comprehensive comprehension (OR=2.89), tertiary education (OR=2.15), and suburban residency (OR=1.87). These findings present the inaugural multivariate data from South Asia, indicating that pharmaceutical care is a controllable factor significantly correlated with enhanced adherence. An important methodological point: our inclusion criterion of 'continuous treatment for ≥6 months' referred to continuous prescribing by physicians, not to patient adherence. The finding that only 45% followed the weekly dosing schedule correctly demonstrates that continuous prescribing does not guarantee adherent behavior. This distinction is critical: patients may collect prescriptions regularly while failing to adhere to complex administration protocols.

Comparison with previous studies

Our 16% optimal adherence rate is lower than Radwan et al. (42% in Palestine) [15] and Vytrisalova et al. (44% in the Czech Republic) [17], likely reflecting healthcare system differences, health literacy levels, and lack of systematic pharmaceutical care in Sri Lanka. However, our median compliance score (4.0/7) aligns with Sewerynek et al. (scores 3-5 in Poland) [13,16]. The pharmacist counseling effect (OR=3.24) supports Lai et al.'s Malaysian intervention study showing pharmaceutical care improved adherence from 43% to 68% [12]. Our study advances this by quantifying independent effects through multivariate adjustment. Our lower adherence rates compared to these studies likely reflect the absence of systematic pharmaceutical care programs in Sri Lankan public hospitals, lower baseline health literacy (26.8% tertiary education in our sample vs. >40% in European studies), resource constraints limiting patient education time, and cultural factors affecting patient-provider communication. The Malaysian study provides the most comparable population context, supporting the generalizability of pharmacist intervention effects in Southeast Asian settings.

Novel contributions

This study makes several important contributions to the existing literature on bisphosphonate adherence. It represents the first multivariate analysis in South Asia to identify independent predictors of alendronate adherence while controlling for potential confounders, addressing a significant gap in regional evidence. The quantification of the pharmacist effect provides robust evidence for policy development, demonstrating that pharmacist counseling increases the odds of optimal adherence more than threefold compared to no instruction (OR=3.24, 95% CI: 1.87-5.61), with this association remaining significant after adjusting for education, residence, and understanding level. The study establishes that the comprehension-adherence relationship operates independently of formal education level, challenging assumptions that educational interventions are only effective for highly educated patients and supporting the development of literacy-appropriate materials for all patient groups. The source-specific comparison of instruction providers reveals that pharmacist counseling is superior to other commonly available sources, including media, leaflets, and physician counseling, providing evidence-based rationale for task-shifting medication education responsibilities to pharmacists. Finally, the adequate statistical power achieved through enrollment of 300 participants enabled robust multivariate modeling and detection of meaningful associations that smaller studies may have missed, strengthening confidence in the validity of the identified predictors.

Clinical and policy implications

Structural modifications at the healthcare system level are essential to enhance the efficacy of bisphosphonate therapy outcomes. Clinical pharmacists ought to be formally incorporated into multidisciplinary osteoporosis clinics, utilizing their proficiency in drug management and patient counseling. Mandatory pharmacist counseling should be implemented at the commencement of bisphosphonate therapy, according to the significant correlation between pharmacist guidance and optimal adherence observed in this study. Healthcare systems must establish suitable reimbursement mechanisms for pharmaceutical care activities to ensure the sustainable implementation of these services, acknowledging the clinical and economic benefits of pharmacist interventions in enhancing medication adherence and potentially decreasing fracture-related expenses.

Patient education tactics necessitate significant improvement to meet the varied demands of the osteoporosis demographic. The creation of pictogram-based educational resources is crucial for engaging low-literacy individuals, who represented a substantial segment of our study sample. Healthcare personnel must employ teach-back techniques to confirm patient comprehension instead of presuming understanding based on passive information reception. Multilingual resources must be accessible to support language variety, and systematic follow-up protocols should be implemented to enhance teaching, evaluate adherence patterns, and tackle developing issues or obstacles during the crucial initial months of therapy.

Clinicians must use a more proactive and systematic strategy for optimizing adherence in ordinary clinical practice. Patient comprehension must be systematically evaluated at each clinical interaction instead of being presumed, employing standardized instruments or structured inquiries to detect knowledge deficiencies. The administration technique must be thoroughly evaluated and rectified, considering the disturbingly low rates of the right technique identified in this study and the probable role of inappropriate administration in adverse consequences. Healthcare practitioners must remain alert for early indicators of non-adherence, including ambiguous gastrointestinal issues, missed appointments, or expressed doubts regarding drug necessity, and undertake prompt interventions to prevent total treatment discontinuation.

Addressing the high prevalence of adverse effects

The occurrence of identified adverse effects in 187 participants (62.3%) requires careful analysis and targeted intervention. This heightened rate presumably signifies multiple contributing factors rather than solely drug-related damage. Insufficient administration techniques appear to be a critical issue, as only 130 participants (43.3%) correctly employed plain water for medication intake, and adherence to other vital administration protocols was similarly deficient. Recall bias inherent in self-reported data may have led to an overestimation of adverse events, particularly concerning common gastrointestinal symptoms that patients attributed to alendronate, regardless of the temporal relationship. The absence of baseline data on pre-existing gastrointestinal disorders impedes the distinction between drug-induced and pre-existing symptoms, hence amplifying the perceived impact of medication-related effects. The notably high hemoptysis rate of 128 patients (42.5%) suggests possible misclassification or misinterpretation of symptoms, given that this is not a widely recognized adverse effect of oral bisphosphonates. The data indicate that comprehensive training on proper administration procedures could substantially reduce preventable adverse effects and improve medication tolerance, hence enhancing long-term adherence and treatment success.

Strengths

The study possesses numerous significant strengths that augment the validity and trustworthiness of the findings. A prospective sample size estimate indicated that 296 participants were necessary, and the study successfully enrolled 300 participants, thus assuring sufficient statistical power to identify significant relationships. Multivariate analysis was utilized to ascertain independent determinants of adherence while accounting for potential confounders, hence enhancing the robustness of the findings. The questionnaire was validated and offered in various languages, guaranteeing cultural relevance and clarity for the broad study population. The elevated response rate of 92.6% reduces non-response bias and improves the sample's representativeness. A thorough evaluation methodology analyzed various dimensions, including sociodemographic parameters, clinical characteristics, knowledge, adherence patterns, instructional delivery, and adverse effects, yielding a comprehensive understanding of the determinants of bisphosphonate utilization. Ultimately, in-person interviews executed by skilled researchers guaranteed data integrity by facilitating immediate clarification of inquiries, validation of answers, and reduction of absent data, which are prevalent issues in self-administered surveys.

Limitations

This study possesses multiple limitations that necessitate careful consideration when evaluating the results. The cross-sectional methodology prevents the establishment of causation between predictors and adherence outcomes, requiring longitudinal research to verify temporal correlations. Self-reported statistics are susceptible to recall bias and social desirability bias, which may lead to an overestimation of adherence levels and comprehension.

Specifically, recall bias regarding instruction receipt presents a significant methodological concern. Of the 143 participants (47.7%) who reported not receiving instructions or could not recall, an unknown proportion may have actually received instructions but forgotten them. This misclassification bias would lead to underestimation of true instruction coverage, potential overestimation of the pharmacist counseling effect if those who recalled were systematically different from those who forgot, and crossover between exposure groups of received versus not received instructions. This limitation is inherent to retrospective self-report and suggests our findings may represent a conservative estimate of instruction provision while highlighting the importance of memorable, reinforced counseling strategies. Additionally, understanding levels were based on self-assessment rather than objective comprehension testing, which may be subject to overestimation due to social desirability bias or metacognitive limitations wherein individuals may not recognize gaps in their own knowledge.

Non-uniform instruction delivery and content among participants who received instructions (n=157) introduces considerable heterogeneity, as the content, quality, and delivery method varied substantially across sources, including pharmacists, physicians, media, leaflets, and unknown sources. This heterogeneity represents real-world practice but introduces several limitations. Content variability means that pharmacist counseling likely included comprehensive dosing protocols, while media messages may have been fragmented or incomplete. Delivery method differences are fundamental, as face-to-face counseling from pharmacists or physicians differs substantially from passive information sources such as leaflets or media in enabling clarification and interaction. Furthermore, it remains unknown whether instructions were one-time events at treatment initiation or were reinforced at follow-up visits, and we could not verify the accuracy or completeness of information received from each source. These variations may confound the source-adherence relationship, though they reflect real-world conditions and support ecological validity. The significant association between pharmacist counseling and adherence despite this heterogeneity suggests a robust effect, though standardized interventions in controlled trials are needed for definitive causality assessment.

Potential interviewer bias exists, though we implemented standardized training and monitoring protocols to minimize inconsistency. The single-center design in a tertiary care environment may restrict the generalizability to primary care or community settings, where the majority of osteoporosis patients are treated. Selection bias is apparent since the sample had a higher level of education than the general population, with 80 participants (27%) possessing university education compared to the national average of 15%, potentially inflating adherence and knowledge results. Adverse occurrences were reported by individuals without medical verification, raising concerns over accuracy and possible misattribution. The lack of objective adherence metrics, such as pharmacy refill data or biochemical indicators, constitutes a substantial constraint, as self-reported adherence generally inflates real medication-taking behavior. The absence of data regarding pre-existing gastrointestinal disorders hinders the differentiation between alendronate-related adverse effects and pre-existing symptoms, potentially inflating the actual burden of drug-related difficulties. Finally, we used WHO international BMI cut-points rather than Asian-specific thresholds, which may not optimally represent metabolic risk in this population, though our analysis showed no significant association between BMI and adherence outcomes regardless of the classification system used.

Future research directions

Future research must emphasize several critical areas to rectify the deficiencies found in this study. Randomized controlled studies are essential to properly evaluate the efficacy of pharmacist-administered treatments on adherence outcomes and clinical objectives. Longitudinal cohort studies must investigate the correlation between adherence patterns and fracture incidence to determine the therapeutic relevance of enhanced compliance. Technology-enhanced interventions, such as mobile applications and SMS reminder systems, merit examination as scalable methods for adherence support. Research on health literacy should concentrate on creating and verifying pictogram-based instructional resources designed for various literacy levels and cultural contexts. Economic assessments of pharmaceutical care programs are crucial to establish cost-effectiveness and facilitate implementation in resource-limited environments. Qualitative studies examining patient barriers, preferences, and experiences with bisphosphonate therapy would yield significant insights to guide patient-centered intervention strategies and enhance treatment results in osteoporosis care. Subgroup analysis comparing knowledge scores between those who received instructions versus those who did not would provide important insights into the impact of patient education on medication knowledge and adherence outcomes.

## Conclusions

Compliance with alendronate dosing instructions is significantly inadequate among postmenopausal women in Sri Lanka, with approximately 48 participants (16%) attaining ideal adherence. Multivariate analysis revealed that pharmacist counseling and comprehensive comprehension are the most significant modifiable determinants of adherence, irrespective of demographic factors. The significant occurrence of gastrointestinal adverse effects (187 participants, 62.3%) and considerable knowledge deficiencies concerning drug-food interactions highlight the pressing necessity for improved patient education.

Healthcare systems must prioritize the implementation of structured pharmaceutical care programs, the development of patient information tailored to health literacy, and the integration of clinical pharmacists into osteoporosis management teams. By targeting these modifiable characteristics, healthcare professionals can markedly enhance treatment adherence, optimize therapeutic results, and diminish fracture risk in postmenopausal women with osteoporosis.

## Appendices

**Table 9:** Knowledge and adherence to alendronate administration questionnaire

Section/Item	Question/Item	Response Options	Correct Answer
SECTION 1: SOCIODEMOGRAPHIC AND HEALTH CHARACTERISTICS			
1.1	What is your current age?	_____ years ☐ 45-54 years ☐ 55-64 years ☐ 65-74 years ☐ ≥75 years	-
1.2	What is your highest level of education completed?	☐ Literate (can read and write) ☐ Primary (Grades 1-5) ☐ Secondary (Grades 6-11/O-Levels/A-Levels) ☐ Diploma☐ Bachelor's degree ☐ Postgraduate degree (Master's/PhD)	-
1.3	What is your total monthly household income?	☐ Low (Less than Rs.40,000) ☐ Moderate (Rs.40,000 - Rs.80,000) ☐ High (More than Rs.80,000)	-
1.4	Where do you currently live?	☐ Urban (within city limits) ☐ Suburban (outskirts of city) ☐ Rural (village/countryside)	-
1.5	Are you currently employed?	☐ Employed (full-time/part-time/self-employed) ☐ Unemployed (retired/homemaker/not working)	-
1.6	Do you smoke or have you ever smoked?	☐ Current smoker ☐ Previous smoker (quit) ☐ Non-smoker (never smoked)	-
1.7	Have you consumed any alcohol in the past month?	☐ Yes ☐ No	-
1.8	How many years ago were you first diagnosed with osteoporosis?	_____ years	-
1.9	Have you experienced any bone fractures since the age of 45?	☐ Yes → If yes, which bones? ___________ ☐ No	-
1.10	If you have experienced any side effects from alendronate, how would you rate their severity?	☐ None (no side effects) ☐ Mild (minimal discomfort, does not interfere with daily activities) ☐ Moderate (noticeable discomfort, some interference with activities) ☐ Severe (significant discomfort, major interference with daily life)	-
1.11	Do you engage in moderate or vigorous physical activity for at least 30 minutes on most days of the week?	☐ Yes ☐ No	-
1.12	Body measurements	Height: _____ cm Weight: _____ kg BMI: _____ kg/m² ☐ Underweight (BMI < 18.5) ☐ Normal weight (BMI 18.5-24.9) ☐ Overweight (BMI 25.0-29.9) ☐ Obese (BMI ≥ 30.0)	-
SECTION 2: KNOWLEDGE OF DRUG-FOOD-MEDICATION INTERACTIONS			
Instructions: For each item, indicate whether you think it is safe to take alendronate together with each substance.			
2.1 Beverages			
2.1.1	Can alendronate be taken with: Tap water	☐ Yes ☐ No	Yes
2.1.2	Can alendronate be taken with: Mineral water	☐ Yes ☐ No	No
2.1.3	Can alendronate be taken with: Milk	☐ Yes ☐ No	No
2.1.4	Can alendronate be taken with: Coffee	☐ Yes ☐ No	No
2.1.5	Can alendronate be taken with: Tea	☐ Yes ☐ No	No
2.1.6	Can alendronate be taken with: Fruit juices (orange, apple, etc.)	☐ Yes ☐ No	No
2.2 Foods			
2.2.7	Can alendronate be taken with: Dairy products (yogurt, cheese, butter)	☐ Yes ☐ No	No
2.2.8	Can alendronate be taken with: Leafy vegetables (spinach, kale)	☐ Yes ☐ No	No
2.3 Medications			
2.3.9	Can alendronate be taken with: Calcium supplements	☐ Yes ☐ No	No
2.3.10	Can alendronate be taken with: Vitamin D supplements	☐ Yes ☐ No	No
2.3.11	Can alendronate be taken with: Antacids (for stomach acidity)	☐ Yes ☐ No	No
2.3.12	Can alendronate be taken with: Cortisone (steroids)	☐ Yes ☐ No	No
2.3.13	Can alendronate be taken with: NSAIDs (painkillers like ibuprofen, aspirin)	☐ Yes ☐ No	No
Knowledge Score: ___/13 (1 point per correct response)			
SECTION 3: ADHERENCE TO DOSING INSTRUCTIONS			
3.1 Current Medication Information	How long have you been taking alendronate?	_____ months	-
3.2 Compliance with Dosing Instructions			
3.2.1	How often do you take alendronate?	☐ Daily (every day) ☐ Weekly (once per week, same day each week) ☐ Monthly ☐ Irregularly/I don't remember ☐ Other: _________	Weekly
3.2.2	What time of day do you usually take alendronate?	☐ First thing in the morning (immediately upon waking) ☐ Morning (but not first thing )☐ Afternoon ☐ Evening ☐ Before bed ☐ Any time/varies	First thing in the morning (immediately upon waking)
3.2.3	What do you drink with alendronate when you take it?	☐ Plain tap water (full glass, 200-250ml) ☐ Small amount of water (less than half glass) ☐ Mineral water ☐ Tea ☐ Coffee ☐ Milk ☐ Juice ☐ Other: _________	Plain tap water (full glass, 200-250ml)
3.2.4	What do you do immediately after taking alendronate?	☐ Remain upright (standing or sitting) for at least 30 minutes ☐ Sit or stand for 10-20 minutes ☐ Lie down immediately ☐ Continue with normal activities (may lie down) ☐ I don't pay attention to this	Remain upright (standing or sitting) for at least 30 minutes
3.2.5	If you miss your scheduled dose of alendronate, what do you do?	☐ Take it as soon as I remember the same day (next morning) ☐ Take it later the same day (afternoon/evening) ☐ Take double dose the next scheduled time ☐ Skip it and resume normal schedule next week ☐ I don't know what to do	Take it the next morning if same day; otherwise skip and resume normal schedule
3.2.6	How long do you wait after taking alendronate before eating your first meal?	☐ At least 30 minutes ☐ 15-20 minutes ☐ Less than 15 minutes ☐ I eat immediately ☐ I don't pay attention to this	At least 30 minutes
3.2.7	How long do you wait after taking alendronate before taking other medications (if any)?	☐ At least 30 minutes ☐ 15-20 minutes ☐ Less than 15 minutes ☐ I take them together ☐ I don't take other medications ☐ I don't pay attention to this	At least 30 minutes
Adherence Score: ___/7 (1 point per correct response) Adherence Category: ☐ Poor (0-2) ☐ Partial (3-5) ☐ Optimal (6-7)			
3.3 Source and Understanding of Instructions			
3.3.8	Did you receive any instructions on how to take alendronate?	☐ Yes → Continue to Questions 9-11 ☐ No → Skip to Section 4 ☐ I don't remember → Skip to Section 4	-
3.3.9	If yes, who provided you with instructions on how to take alendronate? (Select all that apply)	☐ Doctor/Physician ☐ Pharmacist ☐ Nurse ☐ Media (TV, radio, internet, social media) ☐ Package insert/Leaflet ☐ Family member or friend☐ Other: _________ ☐ I don't remember Primary source: _________	-
3.3.10	In what language were the instructions provided?	☐ Sinhala ☐ Tamil ☐ English ☐ Mixed languages ☐ I don't remember	-
3.3.11	How well did you understand the instructions you received?	☐ All (I understood everything completely) ☐ Some (I understood most things, but not everything) ☐ Little (I understood only a small part) ☐ None (I did not understand)	-
SECTION 4: ADVERSE EFFECTS			
Have you experienced any of the following side effects since starting alendronate?			
4.1	General gastrointestinal intolerance (upset stomach, discomfort)	☐ Yes ☐ No	-
4.2	Gastritis (inflammation/burning sensation in stomach)	☐ Yes ☐ No	-
4.3	Peptic ulcer (stomach ulcer)	☐ Yes ☐ No	-
4.4	Dyspepsia (indigestion, bloating, fullness)	☐ Yes ☐ No	-
4.5	Hemoptysis (coughing up blood)	☐ Yes ☐ No	-
4.6	Dysphagia (difficulty swallowing)	☐ Yes ☐ No	-
4.7	Painful or swollen gums	☐ Yes ☐ No	-
4.8	Other side effects (please specify): _________	☐ Yes ☐ No	-
4.9	If you answered "Yes" to any of the above, have these side effects:	☐ Led you to stop taking alendronate temporarily ☐ Led you to stop taking alendronate permanently ☐ Made you consider stopping but you continued ☐ Not affected your medication-taking behavior	-
END OF QUESTIONNAIRE			
Interviewer Notes/Observations: Interviewer Signature: ___________________ Date: ______			
SCORING SUMMARY (For Research Team Only)			
Section 2: Knowledge Score ___/13 Section 3: Adherence Score ___/7 Adherence Category: ☐ Poor ☐ Partial ☐ Optimal Data Entry Date: _____ Data Entry Initials: _____			
